# Prevalence of genital human papillomavirus among rural and urban populations in southern Yunnan province, China

**DOI:** 10.1590/1414-431X20165254

**Published:** 2016-05-31

**Authors:** Z. Baloch, T. Yuan, S. Yindi, Y. Feng, W. Tai, Y. Liu, L. Liu, A. Zhang, B. Wang, X. Wu, X. Xia

**Affiliations:** 1 Faculty of Life Science and Technology, Kunming University of Science and Technology, Kunming, China; 2 The Research Center for Molecular Medicine in Yunnan Province, Kunming, China; 3 The First Hospital in Yunnan Province, Kunming, China; 4 The Second Affiliated Hospital, Kunming Medical University, Kunming, China

**Keywords:** Xishuang Banna, Prevalence, Genotype, Oncogenic, Rural, Urban

## Abstract

This study was designed to investigate and compare the HPV prevalence, genotypes distribution and associated risk factors in rural and urban women living in Xishuang Banna district, in the province of Yunnan. A total of 177 and 190 women from rural and urban areas were engaged, respectively. HPV DNA was amplified using the L1 consensus primers system (MY09/11 and GP5/6) and HPV GenoArray test was conducted for genotyping. Proportions were compared by chi-square test, and logistic regression was used to evaluate risk factors. A total of 54 women were positive for HPV DNA. Among rural women, 23 women were positive for HPV infection, of which 21 showed a single infection and 2 had a multiple infection. HPV-16 (10/23) was the most prevalent genotype followed by HPV-52 (5/23), and HPV-58 (5/23). Urban women had a higher infection rate for overall HPV (31/54) and for multiple genotype infection (8/31). HPV-52 (9/31) was the most prevalent genotype followed by HPV-39 (7/31) and HPV-68 (5/31). The age-specific HPV prevalence was also different between rural and urban women. In urban area, women with age <35 years had the highest HPV prevalence, which declined thereafter as age advanced. However, in rural women the highest HPV prevalence was observed in an older age group (>56 years). Ethnicity, smoking and parity were significantly associated with HPV infection among urban women. Our study demonstrates that HPV prevalence and genotype distribution varies among women from rural and urban areas in the south of Yunnan.

## Introduction

Genital human papillomavirus (HPV) infection is a leading cause of cervical cancer, which is a primary cause of women death in developing regions of the world (1,2). The People's Republic of China is the homeland of approximately 1.3 billion people and is the most densely populated country worldwide. In China, the number of new cervical cancer cases was 78,400 and 20,000 deaths occurred due to cervical cancer in 2010 (3,4). However, the prevalence of HPV infection and its genotypic distribution varies substantially with respect to age, ethnicity, socioeconomic status and geographic location (5,6).

Worldwide, the prevalence of HPV in women with normal cytology is approximately 11–12% with significant regional variation. The highest HPV prevalence has been found in Africa (24%) followed by Latin American (16%), Eastern Europe (21%) and South Eastern Asia (14%) (7). HPV genotype distribution also differs in various regions of the world. In general, HPV-16 is the most frequent in all continents of the world, with some exceptions, whereas the frequency of other genotypes varies from region to region. HPV-18 is the second leading genotype in Europe and in South American continents, while HPV-52 and HPV-58 are more prevalent in the Asian continent (8,9).

Yunnan province is an extension of Tibetan highland with distinctive geographical location, unique landscapes, tremendous differences in elevation, and highly complex topography. The north of the province is dominated by the Himalayan mountain range and has a cold weather, while the equatorial tropic warms up the southern areas. The Xishuang Banna district is located in the southern part of the Yunnan province bordering Burma and Laos (Golden Triangle). Due to the known HPV variation in prevalence and genotype distribution worldwide, and particularly in China, the current study was designed to investigate the prevalence of HPV infection and genotypes distribution in women with normal cervical cytology coming from rural and urban areas of Xishuang Banna district of the Yunnan province.

## Material and Methods

### Study population

There are 56 state-certified ethnic minorities in China, of which 26 are living in the Yunnan province. People from these minority groups prefer to live in community concentrated geographic regions. Xishuang Banna is a Dai concentrated community region. However, ethnic groups like Hani (Hani Zu) are more concentrated in the countryside, while the Han group (largest ethnic group) lives in urban areas throughout China. A total of 368 women (177 rural and 190 urban) were recruited from October to November 2014. Urban women were from Xishuang Banna city and rural women from the Hani village, 60 km southwest of Xishuang Banna. Inclusion criteria were a) the participant should be a permanent resident of the area, b) have above 18 years of age, c) not be pregnant, d) have no history of total uterus or cervical resection, and e) provide written consent. Due to ethnic custom, community meetings were held prior to recruitment of participants in both areas. Interested women were then assigned to appointments at the local community center, where they were individually informed, and written consent was taken. All participants were interviewed by a trained interviewer in a separate room using a standardized questionnaire to gather information on the demographic and social variables, cervical screening and reproductive history, smoking and drinking habits, and sexual behavior. Subsequently, a qualified gynecologist performed the pelvic examination and collected exfoliated cervical cells using a cyto-brush (Hybribio, China).

### Ethical statement

The present study was in line with the Helsinki Declaration and was approved by the ethical review committees of the Kunming University of Science and Technology.

### Cytological analysis

Each sample of exfoliated cervical cells was inserted into a vial with a preservative solution (HBCK-F, Hybribio) and vigorously swirled 10 times. The vials were sent to the Research Center for Molecular Medicine, Kunming, China, for cytological analysis. All cytological slides were individually prepared by two qualified technicians and specimens were classified according to the Bethesda classification system. Smears were free of abnormalities.

### DNA extraction and HPV identification

After collection, cervical samples were immediately transported to the laboratory and stored at -80°C until processing. In detail, the cervical swabs were soaked in 2 mL of a 0.9% solution of sodium chloride for 2–5 h at room temperature and then centrifuged at 10,000 *g* for 10 min. The pelleted cells were re-suspended in 200 μL of TE buffer and digested in a 50-mM solution of proteinase K (Invitrogen, USA) for 5–10 min at 55°C. The DNA was then precipitated with 100% ethanol. Housekeeping *β-globin* gene PCR amplification was done for evaluating the quality of human DNA in all samples (10). Only *β-globin* positive samples were submitted to HPV DNA analysis. MY09/11 and GP5/6 primers were used for amplification of alpha HPV solely (11). DNA from HeLa and Caski cell lines was used as positive control while mixtures without a DNA sample were run as negative controls.

### HPV genotyping

The HPV genotype was determined using the HPV GenoArray test kit (Hybribio) according to the manufacturer's instructions. This test is an L1 consensus primer-based PCR assay that can amplify 21 HPV genotypes, including 15 high risk (HR)-HPV genotypes (16, 18, 31, 33, 35, 39, 45, 51, 52, 53, 56, 58, 59, 66, and 68) and 6 low-risk HPV (LR-HPV) genotypes (6, 11, 42, 43, 44, and CP8304) (12). PCR was performed with a reaction volume of 25 µL that consisted of 5 µL of DNA template, 19.25 µL of the provided master mixture, and 0.75 µL DNA Taq polymerase in a Perkin-Elmer GeneAmp PCR 9700 apparatus (Applied Bio-Systems Inc, USA). The amplification procedure was performed as follows: 9 min of denaturation at 95°C and 40 cycles of 20 s of denaturation at 95°C, 30 s of annealing at 55°C, 30 s of elongation at 72°C, and a final extension for 5 min at 72°C. The amplicon was subsequently denatured and subjected to hybridization. The assay utilized a flow-through hybridization technique that actively directed the targeting molecules towards the immobilized HPV type-specific probes within the membrane fibers, with the complementary molecules retained by the formation of duplexes. After a stringent wash, the hybrids were detected through the addition of streptavidin-horseradish peroxidase conjugate (provided with the kit), which binds to biotinylated PCR products, and a substrate (nitroblue tetrazolium-5-bromo-4-chloro-3-indolylphosphate) that generates a purple precipitate at probe dot. The final results were detected by a colorimetric change on the chip under direct visualization.

### Statistical analysis

HPV prevalence in the rural and urban samples was compared using the chi-square test. The prevalence of HPV infection, presence of single or multiple HPV genotypes, as well as their corresponding 95% confidence intervals (CIs) among rural and urban women were estimated with binomial distribution analysis. The effects of potential risk factors such as ethnic background, age, occupation, education level, marital status, number of sexual partners, Pap test history, smoking and drinking habits, were evaluated using univariate and multivariate logistic regression models; odds ratios and their 95%CIs were calculated. Age-specific prevalence of overall HPV infection, HR-HPV and multiple HPV infections were calculated separately for urban and rural women. A P-value of 0.05 was considered to be statistically significant. The statistical analysis was performed using SPSS version 20.0 (IBM, USA).

## Results

Fifty-four women were positive for HPV infection, of which 44 were positive for a single infection and 10 for multiple genotype infections. HR-HPV genotypes (48/54) were more frequent than low risk genotypes (6/54). HPV-52 was the most frequently detected genotype (14/54) while the distribution of the remaining genotypes was as follows, in a descending order: HPV-16, HPV-58, HPV-39, HPV-68, and HPV-66 (Table 1).

**Table:**
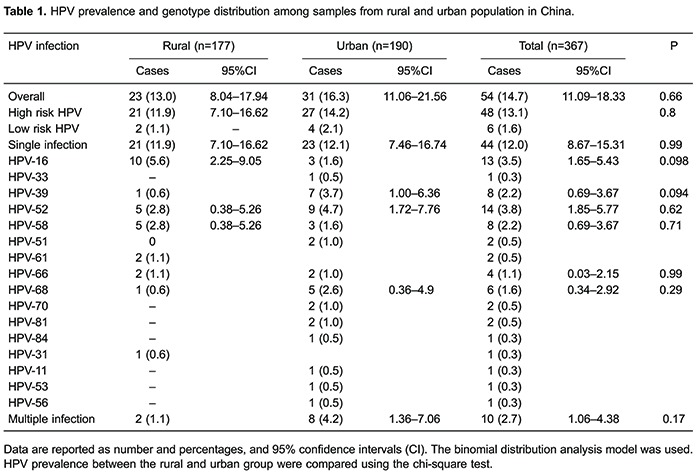

In the rural group, HPV DNA was detected in 23 women, of which 21 were infected with HR-HPV genotypes and 2 with LR-HPV genotype. Eight different HPV genotypes were identified, among which HPV-16 was the most prevalent genotype, while both HPV-52 and HPV-58 were the second most common genotype (Table 1). In the urban population, HPV DNA was detected in 31 participants; 23 women were infected with a single HPV genotype, and 8 with multiple HPV genotypes. The percentage of samples containing at least one HR-HPV genotype was 14.2% in the urban women. A total of 14 HPV genotypes were detected, among which HPV-52 (3.8%) was the most prevalent genotype, followed by HPV-16 (3.5%), and HPV-39 and HPV-68 (both 2.2%) (Table 1).

**Figure 1:**
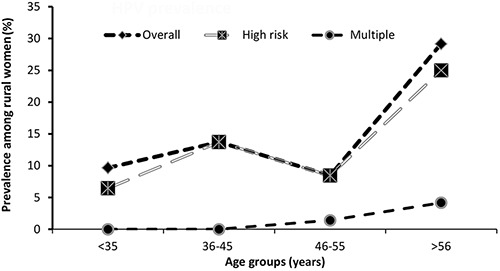
Age-specific prevalence of human papillomavirus (HPV) in 177 women from rural areas in Yunnan, China.

**Figure 2:**
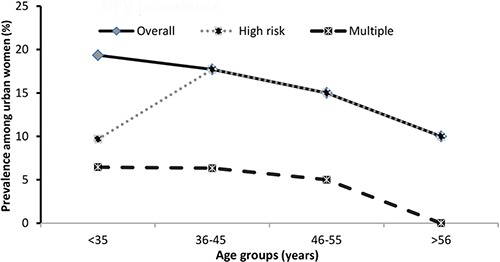
Age-specific prevalence of human papillomavirus (HPV) in 190 women from urban areas in Yunnan, China.

The mean age of participants was 45.87±9.0 (rural) and 44.01±9.6 (urban) years; 48.2% of the women were from rural areas, and 51.8% from urban areas. HPV prevalence among the women from rural areas increased from 9.6% in the younger age group (<35 years) to 29.2% in the older age group (>56 years) (Figure 1). Similar prevalence trends were also observed for HR-HPV, with little variation. The multiple genotype infections yield a single peak in the older age group. In women from urban areas, the younger age group (<35 years) had the highest overall (19.3%), and multiple genotype (6.4%) HPV infection rates, and infection rate declined with increasing age. However, HR-HPV infection showed peak prevalence in the middle age group (36–45 years). Interestingly, all infections in women aged 36–45 and 46–55 years in the urban area were of HR-HPV (Figure 2).

The role of potential risk factors in overall HPV infection is shown in Table 2. Ethnic background was found to be a significant risk factor associated with HPV infection in urban women (P=0.004). Han women (OR=3.33, 95%CI=1.27–8.73) had significantly higher risk for HPV infection compared to Hani and other ethnic women (reference value=1 and OR=0.75, 95%CI=0.27–2.13, respectively). Similarly, women from the urban population who gave birth two or more times (OR=2.45, 95%CI=1.02–5.92, OR=3.16, 95%CI=0.8–11.41) were found to have an increased risk for acquiring HPV infection (P=0.05). On the other hand, 18.4% of women in the urban group were smokers and these women had a higher risk for HPV infection than nonsmokers (OR=3.09, 95%CI=1.32–7.27, P=0.01). Among rural women, 75.7% were from the Hani ethnic group who were more prone to HPV infection than those from Han and from other ethnicity; however, the difference was not significant. Higher number of child births increased the risk for HPV infections in the rural women (OR for 2 births=0.78, 95%CI=0.26–2.34, OR for multiple births=1.44, 95%CI=0.46–4.55); however, this increase was not significant. The other variables evaluated in this study, such as education, occupation, drinking habit, number of sexual partners and past history of Pap smear test were not significant risk factors for HPV infection rate in both groups.

**Table:**
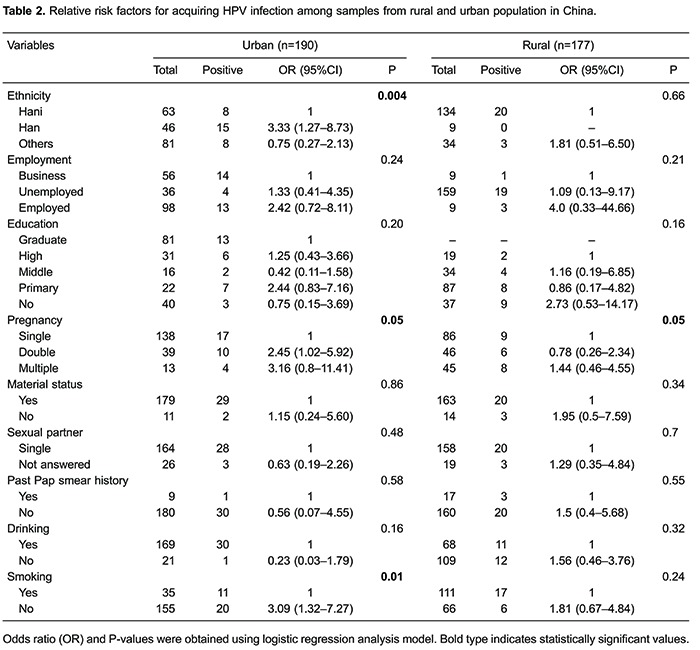

## Discussion

Vaccination against HPV is a possible long-term solution for eradicating cervical cancer in developing countries, particularly in China, where HPV-related infection is a leading cause of morbidity (12%) and mortality (14%) (2). In China, a prophylactic HPV vaccine is undergoing a phase III trial (13). Furthermore, the available HPV vaccine is genotype specific and only controls infections of the genotype for which it was formulated. To maximize the effectiveness of HPV vaccination in China, determining the variation of HPV prevalence and genotype distribution among various populations in different geographical regions of the country is essential. In the current study, 14.7% (54/367) of the participants were positive for HPV infection. This prevalence rate is similar to that reported in a study from Yangcheng County, Shanxi (14.8%) (14), and lower than that reported for South Taiwan (19.3%), and North-western Yunnan (18.4%) (15,16). The frequency of infections with HR-HPV genotypes (13.1%) was slightly higher than that found in a study conducted in Colombian women (11.4%) (17), and Han women (12.6%) from Mojiang county (Yunnan) (18).

In this study, we determined and compared the HPV prevalence in a sample of women living in rural and urban areas in southern parts of Yunnan province. Our study found that women from this rural population in China had a lower HPV prevalence (13.0%) than that of the urban population (16.3%). However, this result is in contrast with other studies from China, where HPV prevalence among rural populations was found to be higher than that of urban populations (13,14,19). However, the HPV prevalence found in rural women is similar to the previously reported worldwide rate of 11–12%. Also, HPV prevalence rate observed among rural women was comparable to data reported in other regions of China, such as Henan province (12.3–13.0%) (20). Our findings for the urban women group (16.3%) was comparable to the results reported from various parts of China (21,22). The difference between HR-HPV prevalence in the rural (11.9%) and urban women (14.2%) was very small. A study from Tibet (23), reported a lower HR-HPV prevalence (7.0%) compared to the findings of this study (11.9%). In studies from Beijing, northern China (24), and Zhejiang province, southeast China (25), the reported HPV prevalence among the urban population was lower (9.9%) than rates found in our study. The most important result of of our study is the high frequency of multiple-genotype infections among the urban population (4.2%). The high percentage of multiple genotypic infections found among the urban population is consistent with data reported from Qujing City, Yunnan province (26). It is well recognized that multiple genotypic infections elevate the risk of cervical cancer (27). Urban and rural populations live in different socioeconomic environments, lifestyles and life standards, which might explain the higher prevalence of overall HPV, oncogenic HPV and multiple-genotype HPV infections in urban populations. Furthermore, sample collection by a single qualified gynaecologist and standardized data analyses minimized errors that could have influenced the results.

HPV-16 was the most prevalent genotype, followed by HPV-52, and HPV-58, in the rural population. These observations are in complete agreement with the study conducted in Shanxi Province, China, in which a relatively high percentage of HPV-16 and then of HPV-58 infection was observed (28), confirming the importance of HPV-58 and HPV-52 infection in Asia (20), particularly in China. In urban women, HPV-52 was the most prevalent, followed by HPV-39 and HPV-68, which is also in agreement with previous reports from China and other Asian countries (29,30). The variation in genotype prevalence may be due to the geographic location of the participants and the biological interactions among genotypes and patients' immune systems (9,31). Interestingly, another oncogenic type, HPV-68, was also common in this population, despite being previously considered an uncommon genotype (9).

The age-specific prevalence curve for rural participants indicated results that were relatively similar to the cross sectional study reported by Franceschi et al. (32) and to data reported from high risk areas of Columbia (17). This pattern is not in agreement with studies conducted in highly developed countries (33). In rural women, the HPV prevalence was inversely correlated with age, with the highest prevalence observed among older age groups. In addition to the overall prevalence of HPV, the age-specific prevalence in urban women also differed from that of rural women. Younger urban women had the highest infection rates, and the prevalence decreased with increasing age. These findings are in line with data reported in previous studies (23), suggesting that younger women are more exposed to HPV infection than older women.

Our study results show that in rural populations, as in Xishuang Banna, HPV DNA prevalence is low in young women (14,16). As most women in rural areas are married and have single sexual partners, the less pronounced second peak of HPV infection in middle age might be due to husbands' extramarital sexual relationships. This risk factor has already been verified in a pooled analysis conducted by the International Agency for Research on Cancer (IARC) (34). The high HPV prevalence among the older age group of rural women might be attributable to weak immune responses for clearing the infection or to a high incidence of HPV infection.

Our results are also consistent with previous studies in respect to the association between HPV DNA detection and ethnic background (16,18,35). HPV prevalence among populations in urban areas was significantly higher among Han than among Hani and other ethnic groups. A similar but non-significant association was also detected in the rural area women. These findings suggest that different ethnic groups may have different risk for HPV infection and development of cervical cancer. It is therefore essential to categorize these population groups and target them for cervical screening program. In this study, we found a clear trend towards an increasing risk for HPV infection with increasing parity among women from both areas. However, only women with two babies from the urban area were at a significantly higher risk of being infected with HPV. In a previous study, women who give birth to a single baby were at lower risk for acquiring HPV infection than multiparous women (36).

The results of previous reports regarding the role of smoking in the acquisition of HPV infection are controversial (20,34). In this study, we found a significantly higher prevalence of HPV infection among smokers in urban women. Most of the rural women were smokers, and HPV infection rate was higher among them, although not significantly. Based on these findings, we speculate that a correlation between smoking and other risk factors such as ethnicity, age, sex, drinking habit, contraceptive use, or sexual behaviors might exist. We will attempt to explain the correlations between potential risk factors in a future study.

Our study demonstrates that HPV prevalence and genotypic distribution varied between women from rural and urban areas, in southern parts of the Yunnan province. These variations highlight the importance of including HPV-52, 58, 39 and 68 in the next generation of HPV vaccines. Further and enlarged monitoring on HPV prevalence is urgently needed for HPV prevention and control strategies in the Yunnan province, China.
